# Radiomics and Machine Learning for Detecting Scar Tissue on CT Delayed Enhancement Imaging

**DOI:** 10.3389/fcvm.2022.847825

**Published:** 2022-05-12

**Authors:** Hugh O'Brien, Michelle C. Williams, Ronak Rajani, Steven Niederer

**Affiliations:** ^1^School of Biomedical Engineering and Imaging Sciences, King's College London, London, United Kingdom; ^2^Centre for Cardiovascular Science, University of Edinburgh, Edinburgh, United Kingdom; ^3^Cardiology Department, Guy's and St Thomas' NHS Foundation Trust, London, United Kingdom

**Keywords:** radiomics analysis, machine learning, delayed enhancement cardiac computed tomography, scar imaging, computed tomography

## Abstract

**Background:**

Delayed enhancement CT (CT-DE) has been evaluated as a tool for the detection of myocardial scar and compares well to the gold standard of MRI with late gadolinium enhancement (MRI-LGE). Prior work has established that high performance can be achieved with manual reading; however, few studies have looked at quantitative measures to differentiate scar and healthy myocardium on CT-DE or automated analysis.

**Methods:**

Eighteen patients with clinically indicated MRI-LGE were recruited for CT-DE at multiple 80 and 100 kV post contrast imaging. Left ventricle segmentation was performed on both imaging modalities, along with scar segmentation on MRI-LGE. Segmentations were registered together and scar regions were estimated on CT-DE. 93 radiomic features were calculated and analysed for their ability to differentiate between scarred and non-scarred myocardium regions. Machine learning (ML) classifiers were trained using the strongest set of radiomic features to classify segments containing scar on CT-DE. Features and classifiers were compared across both tube voltages and combined-energy images.

**Results:**

There were 59 and 51 statistically significant features in the 80 and 100 kV images respectively. Combined-energy imaging increased this to 63 with more features having area under the curve (AUC) above 0.9. The 10 highest AUC features for each image were used in the ML classifiers. The 100 kV images produced the best ML classifier, a support vector machine with an AUC of 0.88 (95% CI 0.87–0.90). Comparable performance was achieved with both the 80 kV and combined-energy images.

**Conclusions:**

CT-DE can be quantitatively analyzed using radiomic feature calculations. These features may be suitable for ML classification techniques to prospectively identify AHA segments with performance comparable to previously reported manual reading. Future work on larger CT-DE datasets is warranted to establish optimum imaging parameters and features.

## Introduction

Imaging of myocardial fibrosis is routinely used for patient diagnosis, prognosis and procedure planning. The clinical gold standard is cardiac magnetic resonance imaging (MRI) with late gadolinium enhancement (LGE) (1). As an alternative to MRI-LGE, delayed enhancement CT (CT-DE) has been proposed in those patients who are unable to undergo MRI scanning owing to availability, cost, claustrophobia, the presence of metallic implants or body size. CT is both cheaper and more widely available than MRI. Even with a delayed enhancement protocol, a cardiac CT scan is much shorter to perform than MRI-LGE, while also providing a higher spatial resolution.

Previous research studies have established that CT-DE can identify myocardial scar using MRI-LGE as a reference standard, in both animal models (2) and patients (3–5). Expert delineated myocardial scar on CT-DE also has a good agreement with invasive electro-mapping of scar (6). Previous studies have shown that visual assessment by expert readers can identify segments containing scar on CT-DE, with accuracy as high as 90% (3). However, there remain questions about the optimal acquisition parameters for CT-DE, and whether using combined-energy imaging can improve scar detection.

Quantitative analysis and the potential for automated analysis of CT-DE has been less well-explored. Radiomic features have been shown to be useful in other quantitative evaluations of CT, such as coronary plaque vulnerability (7) and identification of myocardial infarction (8). One previous study has attempted to assess radiomic features of scar on CT-DE (9), but only used first order parameters and did not use scar confirmation with a reference modality.

The aim of this study was therefore to extract radiomic features which indicate myocardial scar on CT-DE, as determined by the clinical gold standard of MRI-LGE, and to investigate their potential to identify regions with myocardial scar. We also compare multiple energy levels for their suitability for CT-DE scar radiomic analysis.

## Methods

### Study Design

In a single center study we recruited 18 patients who had MRI proven late gadolinium enhancement on MRI imaging performed for clinical indications. The study was approved by the local ethics committee and patients provided written informed consent.

### Image Acquisition

Magnetic resonance imaging was performed as part of the patient's clinical care on a 1.5 Tesla scanner (Siemens Healthineers) at the Edinburgh Heart Center. Sequences were acquired according to the clinical indication, but included localisers, axial HASTE images, and standard breath-held and electrocardiogram-gated CINE sequences. Delayed enhancement images (gradient echo inversion recovery sequences) were performed 10 min after injection of gadolinium contrast agent (0.2 mmol/kg).

Patients underwent cardiac CT imaging using a 320 multidetector scanner (Aquillion One, Canon Medical Systems) at Edinburgh Imaging, University of Edinburgh. Participants with a heart rate of greater than 60 beats/min received intravenous beta blockade prior to CT imaging. Sublingual glyceryl trinitrate was administered prior to CT imaging, unless contraindicated. Electrocardiogram-gated CT was performed 4 min after injection of 100 ml of iodinated contrast (Iomeron 400). Patients underwent CT using four tube voltages in rapid succession (80, 100, 120, and 135 kV). Only the 80 and 100 kV images were used in this study. Tube current was automatically set based on scout image attenuation. Mean radiation dose of 80 kV images was 1.6 ± 0.4 millisievert (mSv) and 100 kV images was 2.6 ± 1.2 mSv (conversion factor 0.028 mSv/mGy.cm).

### Myocardial Scar Region Estimation

The MRI-LGE scans were used to generate image masks for scar regions on the CT-DE scans. Images from both modalities were segmented separately using Siemens Healthineers prototype software, which was previously described by Behar et al. (10). CT-DE segmentation for both 80 and 100 kV as well as MRI CINE segmentation was automatic for the left ventricle. The MRI-LGE scar region segmentation was performed using the same tool. Scar was segmented initially using the full width at half maximum method, with manual corrections by an operator with 3 years experience at this task.

The resulting left ventricle meshes were registered using iterative closest point (ICP) registration performed in custom software using the VTK C++ library (11). Registration was performed in three steps. First the major axis of each mesh is calculated and then registered together. Then the right ventricle insertion points, which are outputted by the segmentation tool, are registered to correctly rotate the LVs from the two modalities. Finally, the whole endocardium meshes are registered to fine tune the registration. The resulting registration transform is applied to the MRI-LGE scar mesh to generate a scar mesh registered to the CT anatomy.

**Figure 1:**
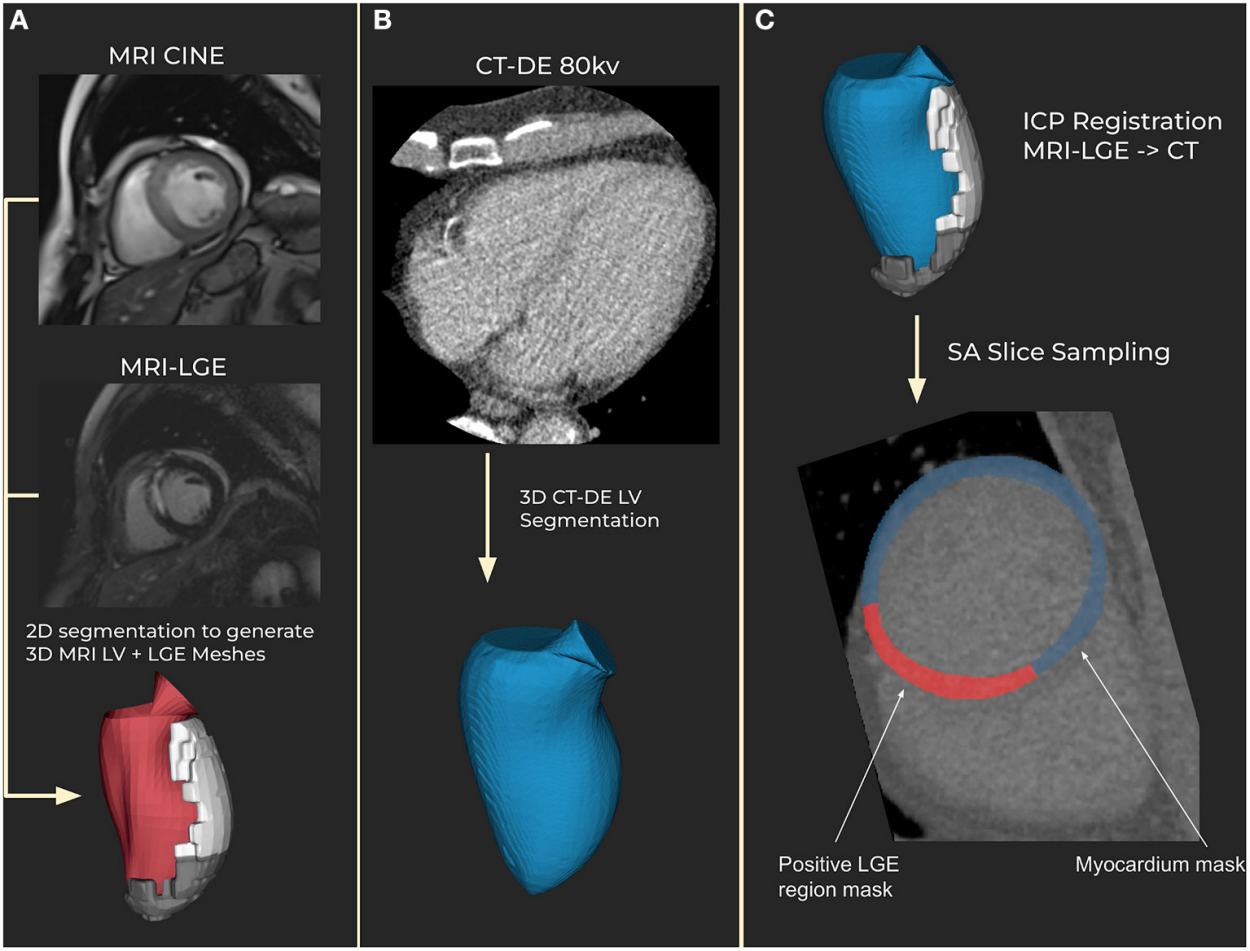
Segmentation and registration. **(A)** Magnetic resonance imaging (MRI) segmentation using CINE MRI for anatomical 3D mesh and MRI late gadolinium enhancement (LGE) for scar mesh. Endocardial (red) and scar meshes (white) shown **(B)** Delayed enhancement computed tomography (CT-DE) segmentation to generate a 3D mesh of left ventricle from CT. CT endocardial mesh is shown in blue. **(C)** CT scar mesh generated by iterative closest point (ICP) registration from the MRI to CT left ventricle anatomical meshes. Applying the resulting transform to the MRI-LGE mesh produces a CT aligned scar mesh. This provides locations to mask for scar on the CT-DE.

Registrations were assessed manually for correctness by matching the aortic valves and apex in both meshes. 60 short-axis slices were then obtained across the CT left ventricle, with myocardium and scar masks calculated from the meshes (Figure 1). The mesh registration and slicing software can be made available on request. All scar regions, regardless of their transmurality, were labeled as transmural to account for differences in phase between the MRI and CT.

### Radiomic Analysis

Radiomic features were calculated using the open-source PyRadiomics package (12) (version 3.0.1). 93 features were calculated for both 80 and 100 kV images. A full list can be found in Supplementary Table S1. These were 18 first order statistics, 24 gray level co-occurrence matrix (GLCM) features, 14 gray level dependence matrix (GLDM) features, 16 gray level size zone matrix (GLSZM) features, 16 gray level run length matrix (GLRLM) features and 5 neighboring gray tone difference matrix (NGTDM) features. Details of the parameters used are included in the Supplementary Methods.

Each feature was tested for discrimination ability using two methods. Statistical difference between scar and non-scar region feature values was determined using a two-sided Student's *t*-test. Linear regression was performed using patient-wise stratified 5-fold cross validation. From this receiver operating characteristic metrics were calculated, with confidence intervals calculated using bootstrapping on 1,000 samples with replacement.

### Combined-Energy Radiomic Analysis

Features were also calculated on combined-energy images, by combining the 100 kV and 80 kV images. Three combinations were considered, with the 100 kV images contributing 40, 50, and 60% to the final image.

Combined-energy images were generated by registering the 100 kV images to the 80 kV using the open source Medical Image Registration Toolkit (MIRTK). The resulting registered 100 kV image was added to corresponding 80 kV images at the three contribution levels to generate the final images.

Features were calculated using the 80 kV segmentation and registered scar meshes from the main analysis. Comparisons with the single-energy images were made by comparing area under receiver operator characteristic curves (AUC) across significant features, which were determined using the same method.

### Per Segment Scar Classification

**Figure 2:**
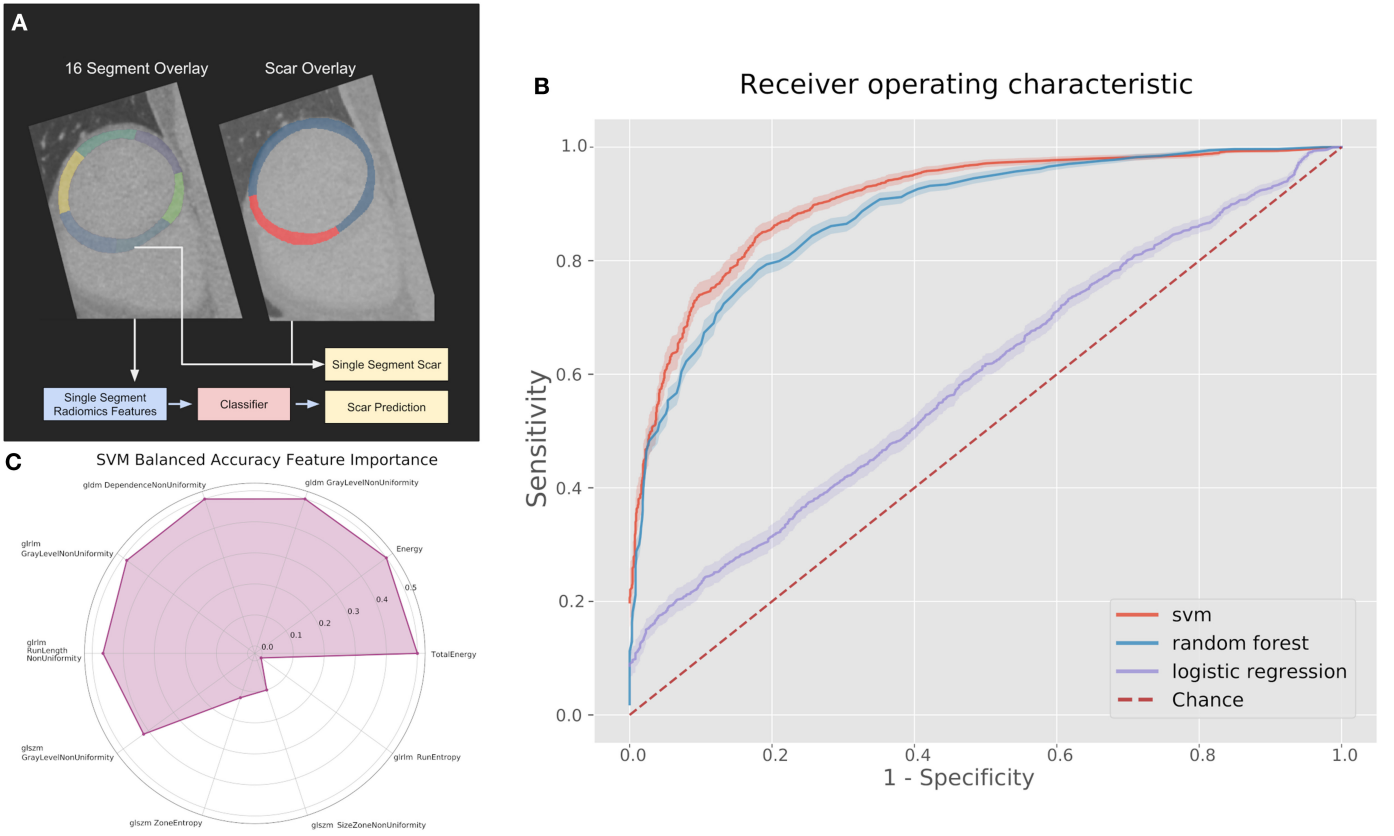
**(A)** Pipeline and results of classification methods using the top 10 radiomic features per myocardial segment. **(B)** Receiver operating characteristics (ROC) curves for classifiers on the 100 kV images in the segmental analysis. **(C)** Permutation feature importance across the 10 features used in the support vector machine (SVM) for the 100 kV segmental classification. GLDM, Gray level dependence matrix; GLRLM, gray level run length matrix; GLSZM, gray level size zone matrix.

To demonstrate possible clinical usage of these radiomic features we trained classifiers to identify myocardial segments as scar or non-scar. Myocardial segments were defined according to the American Heart Association (AHA) 16 segment model. Myocardial scar ground truths were determined by the percentage of the total segment volume which had scar present. Scar thresholds of 10, 20, and 30% total volume were compared as ground truths. This was determined by the MRI-LGE registration and as a proportion of the total region size (Figure 2). This was considered important as a low threshold could produce radiomic features too close to normal myocardium and a higher threshold would miss substantial scar regions. While the previous radiomic analysis compared the whole scar region to healthy myocardium, these classification experiments aim to assess the ability to predict scar using known radiomic features on a meaningful region for evaluating scar burden. Classifiers were compared also across 80 kV, 100 kV, and the best combined-energy level image.

We compared support vector machine (SVM), logistic regression and random forest classifiers implemented with the open source Scikit-learn (13) Python library (version 1.0.1). Parameters were optimized with grid search and 5-fold cross-validation was used. The 10 radiomic features which showed the largest difference between healthy and scared myocardium from the radiomic analysis were used as input features to the classifiers after being scaled to unit length. Feature importance was calculated using the Scikit-learn implementation of permutation importance, which determines the importance of each input feature by re-calculating accuracy metrics after removing each feature.

## Results

### Study Population

Of the 18 available cases, 16 cases had good MRI-LGE segmentation and were suitable for registration. Exclusions were due to a missing or unsuitable short-axis sequence in MRI. 15 cases were used for the 80 kV analysis, with one being excluded due to low contrast between the left ventricle myocardium and the blood pool resulting in a poor wall segmentation. 14 were used for the 100 kV analysis, with poor segmentation results meaning two cases were excluded.

Mean age of the included patients was 62 ± 8.8 and 90% were male. 11 patients had a history of previous myocardial infarction (8 ST elevation and three non-ST elevation myocardial infarction). One patient had previously undergone coronary artery bypass graft surgery.

### Radiomic Analysis

For the 80 kv analysis there were 15 patients with 431 valid slices to perform radiomic analysis and for the 100 kv analysis there were 14 patients with 388 valid slices. The combined-energy analysis included 13 patients with 321 slices.

**Figure 3:**
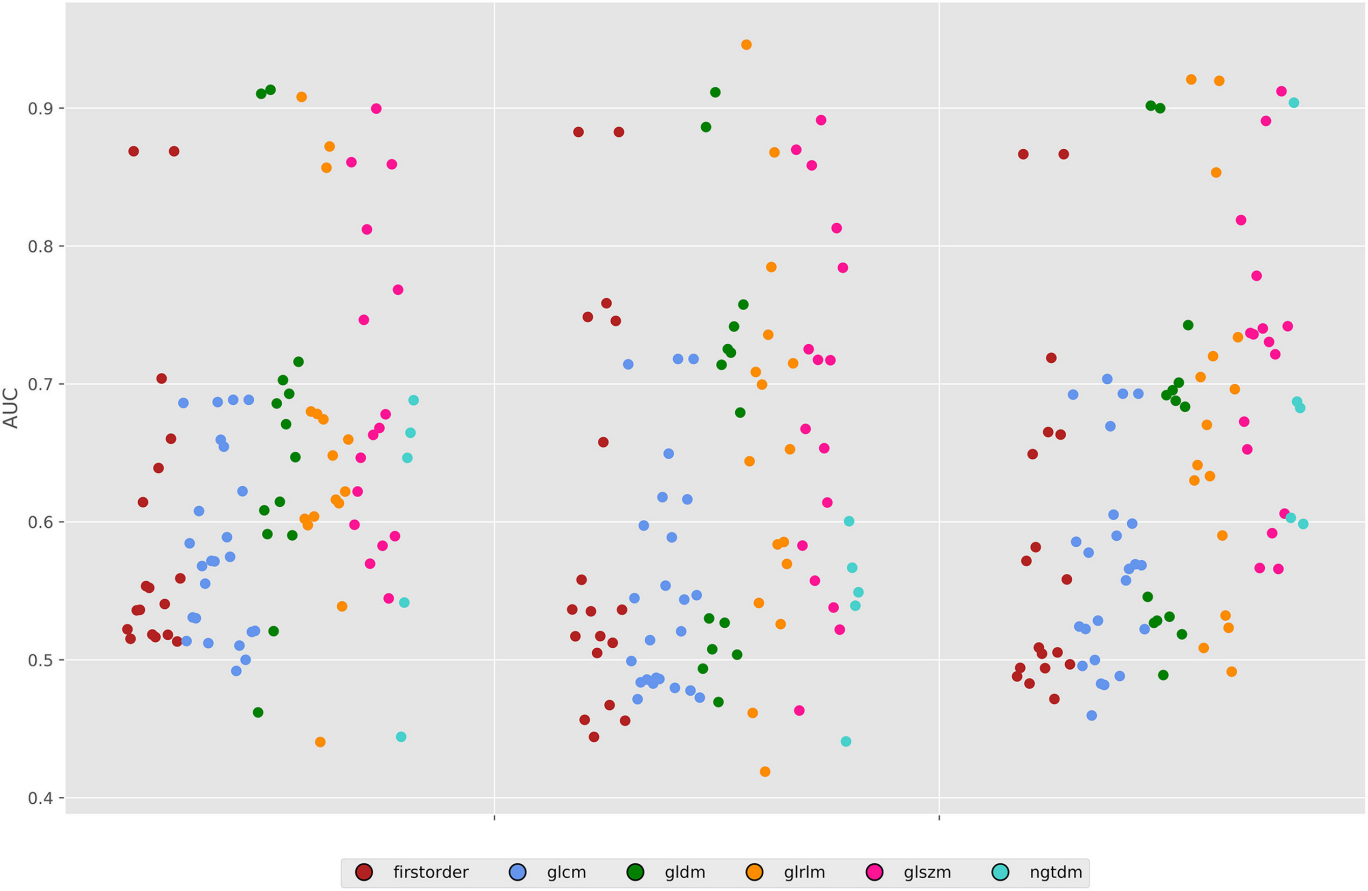
Area under the ROC curves for all features across both energy levels and the 50% combined image. GLDM, Gray level dependence matrix; GLCM, gray Level Co-occurrence matrix; GLRLM, gray level run length matrix; GLSZM, gray level size zone matrix; NGTDM, neighboring gray tone difference matrix.

**Figure 4:**
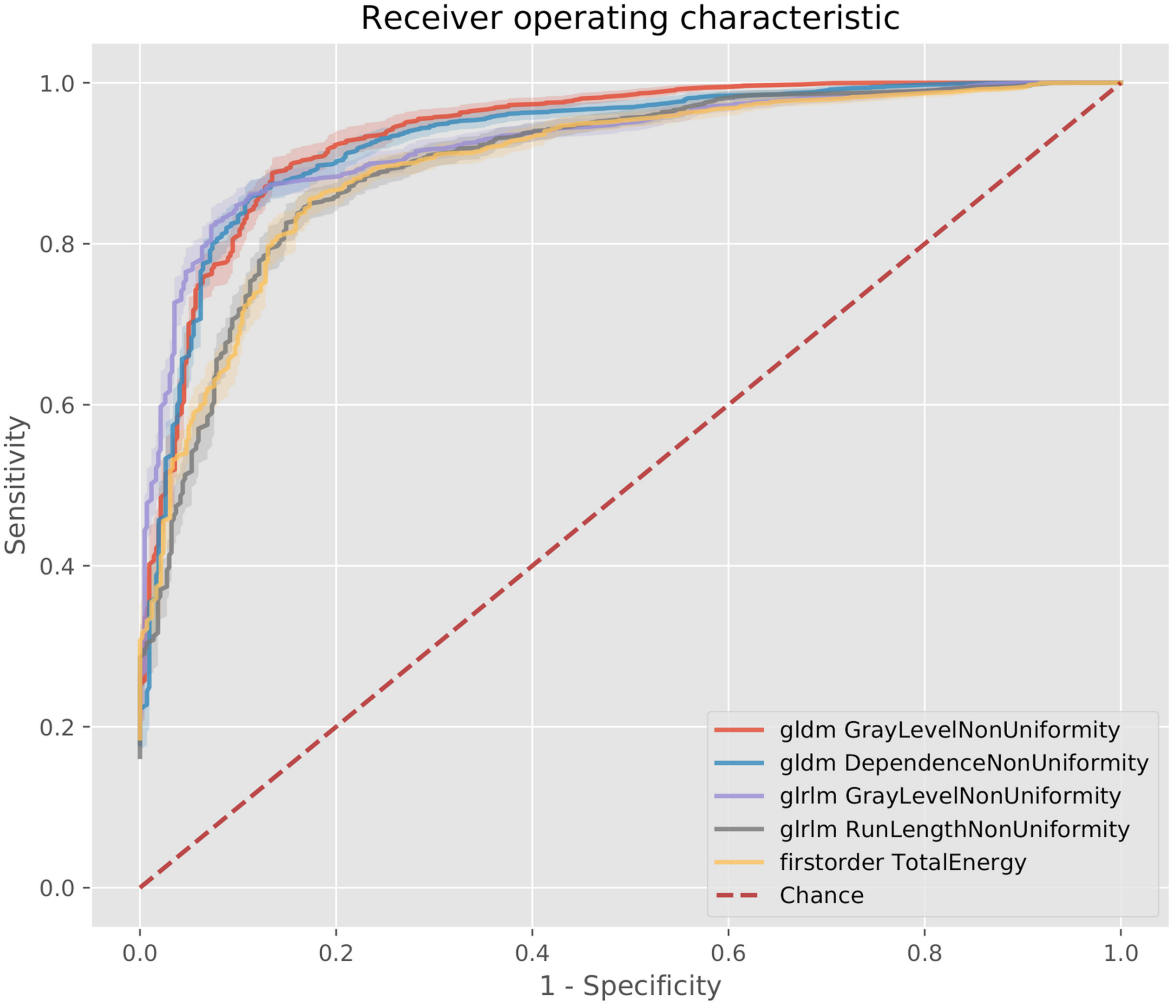
ROC curves for the best 5 AUC values on the 80 kV images. GLDM,Gray level dependence matrix; GLRLM, gray level run length matrix.

**Figure 5:**
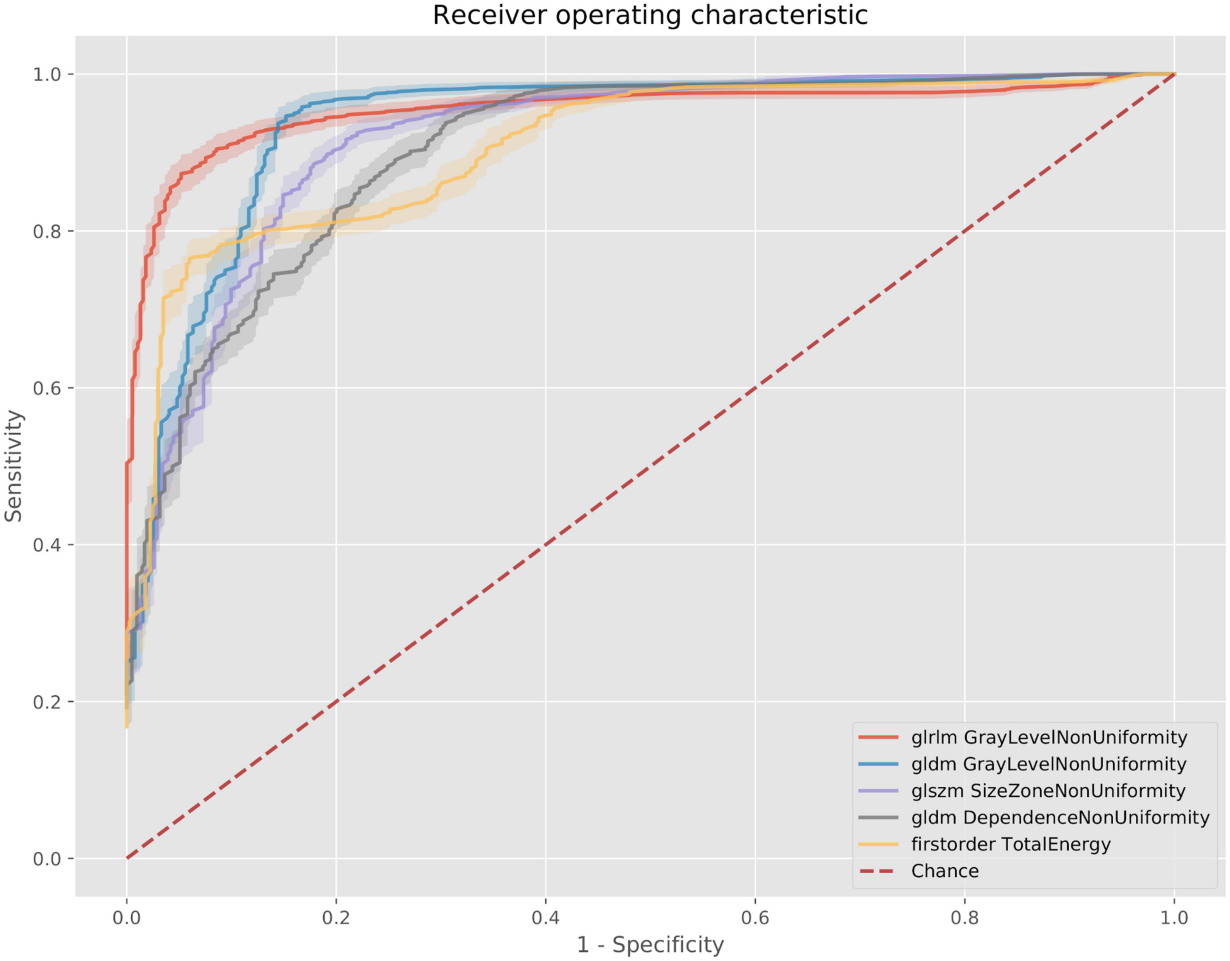
ROC curves for the best 5 AUC values on the 100 kV images. GLDM, Gray level dependence matrix; GLSZM, gray level size zone matrix.

For the 80 kV images, 59 out of the 93 (63%) features were statistically significant predictors of the presence of myocardial scar, whereas 51 (54%) were statistically significant for the 100 kV images. Many of these had low AUCs below 0.7. Above an AUC of 0.7 there were 16 (17%) and 29 (31%) features for the 80 and 100 kV images, respectively. Above 0.8 this was 11 and 10, respectively. There was a clear overlap in features which presented a significant difference between energy levels. Figure 3 displays the AUC values across all features per energy level. The best five AUC values are shown in Figures 4, 5.

The best metric for both 80 and 100 kv was gray level dependence matrix (GLDM) gray level non-uniformity, where the scar region metric was significantly lower than the normal myocardium, indicating greater similarity within scar regions. Other highly significant metrics which were used for the classifier inputs were GLDM dependence non-uniformity, gray level run length matrix (GLRLM) gray level non-uniformity, gray level size zone matrix (GLSZM) size zone non-uniformity, gray level run length matrix (GLRLM) run-length non-uniformity, total energy, energy, GLSZM gray level non-uniformity, GLSZM zone entropy and GLRLM run entropy. Full results for each metric are listed in the Supplementary Methods. These results support a measurable difference in texture in scar overlap regions as compared to normal myocardium.

### Combined-Energy Image Analysis

For the segmental classification analysis there were 1,922 segments (628 with scar) at 80 kv, 1,806 (659 with scar) at 100 kv, and 1,766 (522 with scar) for the combined-energy images.

The 50% 100 kV combined-energy image had the highest number of statistically significant radiomic features to predict scar with 63 significant features (68%). Compared to the other energy levels and combined-energy combinations it also had the highest AUC features, with five above an AUC of 0.9, against three for 80 kV, 2 for 100 kV, and two for both other combined-energy settings. Across high AUC features, the 50% combined-energy images outperformed the other proposed combinations. The top features matched the single-energy images except for NGTDM coarseness, which measures the average difference between a center pixel and its neighborhood. This was significant for all the combined-energy image variations with high AUCs (>0.9); whereas it was only significant for the 100 kV single-energy images with a relatively low AUC (0.6).

### Classification of Scar Using Radiomic Features

Based on radiomic analysis the 10 best features were calculated to identify, *per segment*, CT-DE scar for the 100 kV, 80 kV, and combined-energy 50% images.

The best performing classifier across all images was the SVM. Table 1 shows the AUCs and sensitivities for the SVM at each image level and scar threshold. The 20% scar threshold performed the best for all combinations. The results were similar across image types with slightly higher results for the 100 kV images. SVM was the best performing classification method with a best AUC of 0.88 (95% CI 0.87–0.90) with a sensitivity of 0.79 and specificity of 0.83. The random forest had a comparable AUC with the best being of 0.88 (CI 0.87–0.9) but a worse sensitivity of 0.59 and specificity of 0.92. The logistic regression had a poor AUC with the best being on the 80 kV images at 0.65 (CI 0.62–0.67) with a sensitivity of 0.57 and specificity of 0.61. On this small sample size, these results serve as a proof of concept for the potential to automatically identify scar areas from CT-DE.

**Table 1:** Area under ROC curve, sensitivity and specificity for the classifiers at all three energy levels and three scar thresholds.

	**10% Threshold**			**20% Threshold**			**30% Threshold**		
	**AUC**	**Sensitivity**	**Specificity**	**AUC**	**Sensitivity**	**Specificity**	**AUC**	**Sensitivity**	**Specificity**
**SVM**
80 kV	0.86	0.74	0.82	0.87	0.76	0.82	0.85	0.74	0.85
100 kV	0.88	0.77	0.82	**0.88**	**0.79**	**0.83**	0.88	0.77	0.78
50% combined Image	0.85	0.73	0.85	0.86	0.73	0.86	0.85	0.72	0.88
**Random forest**
80 kV	0.88	0.61	0.9	0.88	0.57	0.94	0.88	0.51	0.94
100 kV	0.87	0.66	0.89	0.88	0.59	0.92	0.88	0.63	0.91
50% combined image	0.88	0.63	0.92	0.87	0.56	0.94	0.87	0.52	0.95
**Logistic regression**
80 kV	0.62	0.54	0.6	0.64	0.57	0.61	0.63	0.54	0.61
100 kV	0.57	0.57	0.5	0.55	0.52	0.53	0.56	0.52	0.54
50% combined image	0.67	0.61	0.62	0.68	0.62	0.63	0.67	0.61	0.63

Figure 2C displays the feature permutation importance for 100 kV images at a 20% threshold, which displays a strong importance across 7 of the 10 included features in terms of changes to balanced accuracy, with the most important feature being first order total energy.

## Discussion

In this study we have demonstrated the ability of radiomic features extracted from CT-DE to predict myocardial scar regions compared to the gold standard assessment of MRI-LGE. Similar features were identified as predictors of myocardial scar and non-scar regions in all examined single-energy and combined-energy images. Good performance was obtained using machine learning methods to classify myocardial segments as scar or non-scar based on three thresholds of scar coverage, with a slightly better performance for the 100 k images.

The ability of visual assessment of CT-DE to act as an alternative to MRI-LGE for expert readers has been established (2, 3, 5). However, quantitative analysis of CT-DE could improve accuracy and repeatability of assessments and reduce the time to report CT-DE. Here we show a path forward for future standardization and automation of scar detection using CT-DE. Our results correspond well-with those of Antunes et al. (9), who found that energy, a first order radiomic statistic, was a statistically significant predictor to identify myocardial scar on CT-DE. However, we have assessed a larger number of radiomic features, and have established the top 10 best radiomic features that can identify scared compared to non-scared myocardium. In future, it may be possible to use these features to prospectively identify scar on CT-DE.

Scar in this study was determined using a 3D mesh registration between MRI-LGE and CT-DE. This provides us with a more accurate delimitation of the scar region compared to previous studies, which used *per segment* classification (2–4). This means that in our radiomic analysis we have calculated the most important features without the overlap of healthy tissue within a segment. This also means that we were able to perform the segmental classification with assessment of different levels of scar volume.

The performance of our method is in line with previously shown manual reading (3). We did not find any clear advantage of combined-energy images or large differences between energy levels in the single-energy images. Previous studies have used either 80 kV or 100 kV images for CT-DE assessment. Visually 80 kV images provides a higher difference between areas of contrast enhancement and non-enhancement compared to 100 kV images, but this is at the expense of image noise. Interestingly we found that the 80 and 100 kV images share similar radiomic features in areas of myocardial scar. However, we showed that there may be a small advantage of 100 kV when using radiomic features. Thus, the choice of 80 or 100 kV for CT-DE images should be made based on the whether visual or quantitative assessment will be performed.

### Study Limitations

This was a single center study with a small number of cases and larger cohort studies with external validation would be required. Our study did not differentiate between ischaemic and non-ischaemic scar or determine the transmurality of myocardial scar.

We used an automated segmentation tool with some user corrections to establish areas of myocardial scar. As we were working with large regions of myocardial scar and radiomic features calculated across them, small segmentation errors would not have greatly affected the results. Nevertheless, more accurate automatic or manual segmentations may produce stronger radiomic features and resulting classifiers. While segmental analyses are clinically useful, with additional datasets it may be possible to produce a more specific classification of scar regions. Convolutional neural networks have been shown capable of this task for MRI with LGE (14) and could be applied here instead of calculating radiomic features.

## Conclusion

This study showed that CT-DE can identify myocardial scar using radiomic features and machine learning methods, with good accuracy compared to the gold standard of MRI-LGE. Further large prospective studies are required to evaluate the use of this technique in clinical practice.

## Data Availability Statement

The data analyzed in this study was subject to the following licenses/restrictions: The data used for this study was imaging data from an NHS hospital for which ethics was acquired for specific use in this study. The mesh registration and slice generation software can be made available on request to the authors. Requests to access these datasets should be directed to hugh.o'brien@kcl.ac.uk.

## Ethics Statement

The studies involving human participants were reviewed and approved by the local ethics committee. The patients/participants provided their written informed consent to participate in this study.

## Author Contributions

HO'B, MW, and SN: conception and design. HO'B and MW: analysis and interpretation of data. All authors were involved in drafting of the manuscript or revising it for content and approved the final manuscript submitted.

## Funding

MW (FS/ICRF/20/26002) was supported by the British Heart Foundation. HO'B would like to acknowledge funding from the EPSRC Centre for Doctoral Training in Medical Imaging (EP/L015226/1). The study was funded by Edinburgh & Lothians Health Foundation (49–187), and by core funding from the Wellcome/EPSRC Centre for Medical Engineering [WT203148/Z/16/Z]. For the purpose of Open Access, the author has applied a CC BY public copyright license to any Author Accepted Manuscript version arising from this submission. The software used for segmenting MRI and CTA datasets was provided by Siemens Healthineers. The software is currently in a prototype stage and commercial availability can't be guaranteed.

## Conflict of Interest

MW - Speaker bureau for Canon Medical Systems. The remaining authors declare that the research was conducted in the absence of any commercial or financial relationships that could be construed as a potential conflict of interest.

## Publisher's Note

All claims expressed in this article are solely those of the authors and do not necessarily represent those of their affiliated organizations, or those of the publisher, the editors and the reviewers. Any product that may be evaluated in this article, or claim that may be made by its manufacturer, is not guaranteed or endorsed by the publisher.
