# Supplementary material for: Radiomics and Machine Learning for Detecting Scar Tissue on CT Delayed Enhancement Imaging
Source: Front Cardiovasc Med. 2022 May 12;9:847825. doi: 10.3389/fcvm.2022.847825 (PMC9133416; doi:10.3389/fcvm.2022.847825)
Supplement: Supplementary file 1 [file Table_1.DOCX]

| **First Order** | | |
| --- | --- | --- |
| 10th Percentile | Maximum | Robust Mean  Absolute Deviation |
| 90th Percentile | Mean Absolute Deviation | Root Mean Squared |
| Energy | Mean | Skewness |
| Entropy | Median | Total Energy |
| Interquartile Range | Minimum | Uniformity |
| Kurtosis | Range | Variance |
| **Gray Level Co-occurrence Matrix** | | |
| Autocorrelation | Difference Variance | Joint Average |
| Cluster Prominence | Inverse Difference | Joint Energy |
| Cluster Shade | Inverse Difference Moment | Joint Entropy |
| Cluster Tendency | Inverse Difference Moment  Normalized | Maximal Correlation Coefficient |
| Contrast | Inverse Difference Normalized | Maximum Probability |
| Correlation | Informational Measure of  Correlation 1 | Sum Average |
| Difference Average | Informational Measure of  Correlation 2 | Sum Entropy |
| Difference Entropy | Inverse Variance | Sum Squares |
| **Gray Level Dependence Matrix** | | |
| Dependence Entropy | Gray Level Variance | Low Gray Level Emphasis |
| Dependence Non-Uniformity | High Gray Level Emphasis | Small Dependence Emphasis |
| Dependence Non-Uniformity  Normalized | Large Dependence Emphasis | Small Dependence  High Gray Level Emphasis |
| Dependence Variance | Large Dependence  High Gray Level Emphasis | Small Dependence  Low Gray Level Emphasis |
| Gray Level Non-Uniformity | Large Dependence  Low Gray Level Emphasis |  |
| **Gray Level Run Length Matrix** | | |
| Gray Level Non-Uniformity | Long Run Low  Gray Level Emphasis | Run Variance |
| Gray Level Non-Uniformity  Normalized | Low Gray Level  Run Emphasis | Short Run Emphasis |
| Gray Level Variance | Run Entropy | Short Run High  Gray Level Emphasis |
| High Gray Level  Run Emphasis | Run Length Non-Uniformity | Short Run Low  Gray Level Emphasis |
| Long Run Emphasis | Run Length Non-Uniformity  Normalized |  |
| Long Run High  Gray Level Emphasis | Run Percentage |  |
| **Gray Level Size Zone Matrix** | | |
| Gray Level Non-Uniformity | Large Area Low  Gray Level Emphasis | Small Area Low  Gray Level Emphasis |
| Gray Level Non-Uniformity  Normalized | Low Gray Level  Zone Emphasis | Zone Entropy |
| Gray Level Variance | Size Zone Non-Uniformity | Zone Percentage |
| High Gray Level  Zone Emphasis | Size Zone Non-Uniformity  Normalized | Zone Variance |
| Large Area Emphasis | Small Area Emphasis |  |
| Large Area High  Gray Level Emphasis | Small Area High  Gray Level Emphasis |  |
| **Neighbouring Gray Tone Difference Matrix** | | |
| Busyness | Complexity | Strength |
| Coarseness | Contrast |  |

Table S1: Full list of radiomic Features
