# Supplementary material for: Radiomics and Machine Learning for Detecting Scar Tissue on CT Delayed Enhancement Imaging
Source: Front Cardiovasc Med. 2022 May 12;9:847825. doi: 10.3389/fcvm.2022.847825 (PMC9133416; doi:10.3389/fcvm.2022.847825)
Supplement: Supplementary file 2 [file Table_2.DOCX]

## Radiomic Analysis Parameters

The radiomic analysis was performed using the version 3.0.1 PyRadiomics package. The formulas for each feature (listed in Table S1) and the source code is available at <https://pyradiomics.readthedocs.io/en/v3.0.1/> . Here we detail the parameters used to perform the analyses.

- A bin width of 25 was used with no fixed bin count for metrics that required grey value discretization. Future work could look at the effect of varying over this parameter for further optimisation.
- The neighbouring gray tone difference matrix (NGTDM) was calculated with a distance of 1 for neighbours
- The gray level run length matrix (GLRLM) and gray level co-occurrence matrix (GLCM) were calculated without any norm weighting, meaning the values were calculated for each angle separately before calculating a mean
- The symmetrical GLCM was used, which is the default in PyRadiomics

No resampling or other operations were required to match the mask and images as they were generated by our VTK slicing code post-registration in identical dimensions.

## Feature Statistics

Here we present a full listing of radiomic statistics for 100kV and 80kV images for comparison in future analyses:

| **Statistic** | **Value** | **p-value** | **ROC** |
| --- | --- | --- | --- |
| **100kV** | | | |
| firstorder_10Percentile | -0.881541636 | 0.3782983969 | 0.5364777075 |
| firstorder_90Percentile | -0.7096769338 | 0.4781182654 | 0.5170647784 |
| firstorder_Energy | 21.46342679 | 0 | 0.8826588904 |
| firstorder_Entropy | 2.171593689 | 0.030199563 | 0.5580295462 |
| firstorder_InterquartileRange | -2.660293159 | 0.0080401304 | 0.4564944468 |
| firstorder_Kurtosis | 5.440411892 | 0.00000008253 | 0.7485652035 |
| firstorder_Maximum | 1.323220576 | 0.1861534392 | 0.5351491923 |
| firstorder_MeanAbsoluteDeviation | -0.708084856 | 0.4791303519 | 0.4440894356 |
| firstorder_Mean | -0.3874549015 | 0.6985260306 | 0.5049553619 |
| firstorder_Median | -0.5752609269 | 0.5652820325 | 0.5171743809 |
| firstorder_Minimum | -7.470160775 | 0 | 0.6578043628 |
| firstorder_Range | 7.502721082 | 0 | 0.7585224253 |
| firstorder_RobustMeanAbsoluteDeviation | -1.52952273 | 0.1266325055 | 0.467159103 |
| firstorder_RootMeanSquared | -0.6068810248 | 0.5441081608 | 0.5123086938 |
| firstorder_Skewness | -8.894903171 | 0 | 0.7457022532 |
| firstorder_TotalEnergy | 21.46342679 | 0 | 0.8826588904 |
| firstorder_Uniformity | -1.08508818 | 0.2782397381 | 0.5363083218 |
| firstorder_Variance | -0.8706393527 | 0.3842504767 | 0.4558600808 |
| glcm_Autocorrelation | 5.854152038 | 0.000000007717 | 0.7142363694 |
| glcm_ClusterProminence | 0.01102994028 | 0.991202402 | 0.4990833245 |
| glcm_ClusterShade | -1.097923295 | 0.2726078405 | 0.5446978956 |
| glcm_ClusterTendency | -0.9324729601 | 0.3514160492 | 0.4714568498 |
| glcm_Contrast | -0.9740576586 | 0.330369109 | 0.4836858327 |
| glcm_Correlation | 2.79530531 | 0.005316709662 | 0.597346955 |
| glcm_DifferenceAverage | -0.9034066934 | 0.3666201973 | 0.4857118185 |
| glcm_DifferenceEntropy | 0.4993582932 | 0.6176733055 | 0.5142748964 |
| glcm_DifferenceVariance | -0.8520256538 | 0.3944888277 | 0.4827824423 |
| glcm_Id | 0.5607324502 | 0.5751512197 | 0.4869805505 |
| glcm_Idm | 0.5791675522 | 0.5626543238 | 0.4860838027 |
| glcm_Idmn | 5.013017012 | 0.00000068883 | 0.6179389414 |
| glcm_Idn | 4.389748703 | 0.000013195471 | 0.5537915825 |
| glcm_Imc1 | -6.510602493 | 0.000000000141 | 0.6494911787 |
| glcm_Imc2 | 4.472623167 | 0.000008931523 | 0.5887846742 |
| glcm_InverseVariance | -0.03589805656 | 0.9713733571 | 0.4796604315 |
| glcm_JointAverage | 8.148903969 | 0 | 0.718115634 |
| glcm_JointEnergy | -0.6897710408 | 0.4905565023 | 0.5206517696 |
| glcm_JointEntropy | 1.477861166 | 0.139865315 | 0.5436815815 |
| glcm_MCC | 4.281008487 | 0.000021008044 | 0.6162982251 |
| glcm_MaximumProbability | 0.8695225797 | 0.384853613 | 0.4777008715 |
| glcm_SumAverage | 8.148903969 | 0 | 0.718115634 |
| glcm_SumEntropy | 1.632305335 | 0.103035315 | 0.5468633755 |
| glcm_SumSquares | -0.9396321318 | 0.3477332749 | 0.4726193007 |
| gldm_DependenceEntropy | -0.4013080718 | 0.6883059038 | 0.4935965565 |
| gldm_DependenceNonUniformity | 21.42316466 | 0 | 0.8862790945 |
| gldm_DependenceNonUniformityNormalized | 2.043925103 | 0.04130093992 | 0.5300178021 |
| gldm_DependenceVariance | -0.5854455056 | 0.5584193909 | 0.507665533 |
| gldm_GrayLevelNonUniformity | 25.22392486 | 0 | 0.9114544585 |
| gldm_GrayLevelVariance | -0.9240075097 | 0.3558037959 | 0.469384366 |
| gldm_HighGrayLevelEmphasis | 5.550952416 | 0.000000041348 | 0.7138776703 |
| gldm_LargeDependenceEmphasis | 2.577347844 | 0.01014679974 | 0.5268360081 |
| gldm_LargeDependenceHighGrayLevelEmphasis | 6.602347689 | 0 | 0.7252896163 |
| gldm_LargeDependenceLowGrayLevelEmphasis | -6.96895074 | 0 | 0.7227122967 |
| gldm_LowGrayLevelEmphasis | -8.206333438 | 0 | 0.7416369965 |
| gldm_SmallDependenceEmphasis | -1.263550273 | 0.2067831811 | 0.5037397704 |
| gldm_SmallDependenceHighGrayLevelEmphasis | 3.543905213 | 0.000418386704 | 0.6792565629 |
| gldm_SmallDependenceLowGrayLevelEmphasis | -11.3191361 | 0 | 0.7575459666 |
| glrlm_GrayLevelNonUniformity | 31.23640309 | 0 | 0.9459892125 |
| glrlm_GrayLevelNonUniformityNormalized | -4.561861127 | 0.000005905987 | 0.6439645552 |
| glrlm_GrayLevelVariance | -0.08651922357 | 0.9310771908 | 0.4614664151 |
| glrlm_HighGrayLevelRunEmphasis | 4.735149045 | 0.000002645811 | 0.708663248 |
| glrlm_LongRunEmphasis | 2.955467909 | 0.003237585345 | 0.5412039005 |
| glrlm_LongRunHighGrayLevelEmphasis | 5.6527463 | 0.00000002686 | 0.6995562759 |
| glrlm_LongRunLowGrayLevelEmphasis | 0.8256464015 | 0.4093414197 | 0.4189273568 |
| glrlm_LowGrayLevelRunEmphasis | -9.037206756 | 0 | 0.735671963 |
| glrlm_RunEntropy | 15.889202 | 0 | 0.7847606016 |
| glrlm_RunLengthNonUniformity | 12.74025384 | 0 | 0.8678858008 |
| glrlm_RunLengthNonUniformityNormalized | -4.606803574 | 0.00000482279 | 0.5836765331 |
| glrlm_RunPercentage | -2.49899042 | 0.01266841832 | 0.5258794771 |
| glrlm_RunVariance | 4.389178767 | 0.000013110795 | 0.5853969604 |
| glrlm_ShortRunEmphasis | 1.87064731 | 0.06177204011 | 0.5695344883 |
| glrlm_ShortRunHighGrayLevelEmphasis | 2.667772609 | 0.00779557703 | 0.6526464024 |
| glrlm_ShortRunLowGrayLevelEmphasis | -10.96742114 | 0 | 0.7149471251 |
| glszm_GrayLevelNonUniformity | 23.32873023 | 0 | 0.8698154692 |
| glszm_GrayLevelNonUniformityNormalized | -0.8060782536 | 0.420446925 | 0.4632433043 |
| glszm_GrayLevelVariance | 1.884397354 | 0.05989609041 | 0.5828329259 |
| glszm_HighGrayLevelZoneEmphasis | 4.407201385 | 0.000011997102 | 0.6674062068 |
| glszm_LargeAreaEmphasis | 4.47501426 | 0.000009998028 | 0.7251766925 |
| glszm_LargeAreaHighGrayLevelEmphasis | 8.747399645 | 0 | 0.8584732703 |
| glszm_LargeAreaLowGrayLevelEmphasis | 3.141023356 | 0.001809879301 | 0.5573453608 |
| glszm_LowGrayLevelZoneEmphasis | -11.48081983 | 0 | 0.7174746254 |
| glszm_SizeZoneNonUniformity | 13.37406716 | 0 | 0.8912809544 |
| glszm_SizeZoneNonUniformityNormalized | -6.210914692 | 0.000000001058 | 0.6533936922 |
| glszm_SmallAreaEmphasis | 5.346987614 | 0.000000118553 | 0.6140463918 |
| glszm_SmallAreaHighGrayLevelEmphasis | 5.554761417 | 0.000000040087 | 0.717205601 |
| glszm_SmallAreaLowGrayLevelEmphasis | -4.466276567 | 0.00000945039 | 0.5379224678 |
| glszm_ZoneEntropy | 14.72364576 | 0 | 0.8130048358 |
| glszm_ZonePercentage | -1.916471115 | 0.0556866227 | 0.5218806462 |
| glszm_ZoneVariance | 6.096546884 | 0.000000002614 | 0.7842756935 |
| ngtdm_Busyness | -0.864253568 | 0.3879801096 | 0.4408212881 |
| ngtdm_Coarseness | -2.915111924 | 0.003692018204 | 0.6004888936 |
| ngtdm_Complexity | 0.1576757004 | 0.8747595431 | 0.566764534 |
| ngtdm_Contrast | -1.781270664 | 0.07539153948 | 0.5392509831 |
| ngtdm_Strength | -0.4857491047 | 0.6272862339 | 0.5489757147 |
|  |  |  |  |
|  |  |  |  |
| **80kV** | | | |
| firstorder_10Percentile | -1.236823467 | 0.2164917202 | 0.5221252039 |
| firstorder_90Percentile | -0.4482582754 | 0.6540797725 | 0.5152588541 |
| firstorder_Energy | 22.438033 | 0 | 0.8686968739 |
| firstorder_Entropy | 2.891002158 | 0.003942700106 | 0.5358632867 |
| firstorder_InterquartileRange | 0.9922036013 | 0.3213973067 | 0.5362347317 |
| firstorder_Kurtosis | 2.335068508 | 0.0197707886 | 0.6142839455 |
| firstorder_Maximum | 3.056771995 | 0.002306855166 | 0.5534719344 |
| firstorder_MeanAbsoluteDeviation | 2.173314935 | 0.03003796867 | 0.5521934098 |
| firstorder_Mean | -0.7579840007 | 0.4486687191 | 0.5183165465 |
| firstorder_Median | -0.535888699 | 0.5921744069 | 0.5164754712 |
| firstorder_Minimum | -7.135468136 | 0 | 0.6390254144 |
| firstorder_Range | 7.85020118 | 0 | 0.7039502371 |
| firstorder_RobustMeanAbsoluteDeviation | 1.109690792 | 0.2674620881 | 0.5404417504 |
| firstorder_RootMeanSquared | -0.7372384418 | 0.4611789122 | 0.5181335156 |
| firstorder_Skewness | -4.318735558 | 0.000017554901 | 0.6602731467 |
| firstorder_TotalEnergy | 22.438033 | 0 | 0.8686968739 |
| firstorder_Uniformity | -2.086993387 | 0.03719951736 | 0.5132697391 |
| firstorder_Variance | 1.469889166 | 0.1419581232 | 0.5590409182 |
| glcm_Autocorrelation | 3.882990372 | 0.000111143323 | 0.6862177745 |
| glcm_ClusterProminence | 0.4901762377 | 0.6241362958 | 0.5135873515 |
| glcm_ClusterShade | -0.949286425 | 0.3427464073 | 0.5844795194 |
| glcm_ClusterTendency | 1.356531202 | 0.1752862082 | 0.5307653383 |
| glcm_Contrast | -0.3847893741 | 0.7004900953 | 0.5301570297 |
| glcm_Correlation | 7.021141566 | 0 | 0.6078536399 |
| glcm_DifferenceAverage | -1.265961486 | 0.2058783324 | 0.5680013566 |
| glcm_DifferenceEntropy | -0.3511261458 | 0.7255826555 | 0.5552322608 |
| glcm_DifferenceVariance | -0.2219234331 | 0.8244271027 | 0.5120638885 |
| glcm_Id | 1.4712825 | 0.1415955653 | 0.5717158069 |
| glcm_Idm | 1.458833778 | 0.1449908223 | 0.5713658949 |
| glcm_Idmn | 10.20357636 | 0 | 0.6868799156 |
| glcm_Idn | 7.681725148 | 0 | 0.6595221817 |
| glcm_Imc1 | -7.960006188 | 0 | 0.6545372818 |
| glcm_Imc2 | 5.114993915 | 0.000000394126 | 0.5889045602 |
| glcm_InverseVariance | -1.545146093 | 0.1226956034 | 0.5746927504 |
| glcm_JointAverage | 6.01108162 | 0.000000002732 | 0.6884787442 |
| glcm_JointEnergy | -1.495806141 | 0.1350885707 | 0.4919304913 |
| glcm_JointEntropy | 2.125078488 | 0.03387740721 | 0.5103250951 |
| glcm_MCC | 7.421047256 | 0 | 0.622216181 |
| glcm_MaximumProbability | -1.188147711 | 0.2351083165 | 0.5000161498 |
| glcm_SumAverage | 6.01108162 | 0.000000002732 | 0.6884787442 |
| glcm_SumEntropy | 2.431913938 | 0.01523185028 | 0.5202249127 |
| glcm_SumSquares | 1.018678233 | 0.3086422318 | 0.5209678027 |
| gldm_DependenceEntropy | 0.2790033723 | 0.7803100209 | 0.4618622854 |
| gldm_DependenceNonUniformity | 17.6620745 | 0 | 0.9103956159 |
| gldm_DependenceNonUniformityNormalized | 2.257027705 | 0.02425656403 | 0.6083731246 |
| gldm_DependenceVariance | -2.679111681 | 0.007525817245 | 0.5911413052 |
| gldm_GrayLevelNonUniformity | 22.8175702 | 0 | 0.9132541276 |
| gldm_GrayLevelVariance | 1.009794792 | 0.3128776459 | 0.5207632388 |
| gldm_HighGrayLevelEmphasis | 3.855782909 | 0.00012402016 | 0.6858463294 |
| gldm_LargeDependenceEmphasis | 4.023639844 | 0.000062511603 | 0.6145854081 |
| gldm_LargeDependenceHighGrayLevelEmphasis | 4.394843111 | 0.000012483495 | 0.7027955276 |
| gldm_LargeDependenceLowGrayLevelEmphasis | -7.672144664 | 0 | 0.6708243388 |
| gldm_LowGrayLevelEmphasis | -8.999497189 | 0 | 0.6928714854 |
| gldm_SmallDependenceEmphasis | -3.95074114 | 0.000085865867 | 0.5902638336 |
| gldm_SmallDependenceHighGrayLevelEmphasis | 3.155068966 | 0.001660877268 | 0.6469280419 |
| gldm_SmallDependenceLowGrayLevelEmphasis | -10.45397862 | 0 | 0.7160867997 |
| glrlm_GrayLevelNonUniformity | 24.23158543 | 0 | 0.9081077298 |
| glrlm_GrayLevelNonUniformityNormalized | -5.1910846 | 0.000000269112 | 0.6022012155 |
| glrlm_GrayLevelVariance | 2.295666105 | 0.02193543499 | 0.59763621 |
| glrlm_HighGrayLevelRunEmphasis | 3.710967457 | 0.00021983912 | 0.6800539403 |
| glrlm_LongRunEmphasis | 2.596113746 | 0.009660764301 | 0.6039050177 |
| glrlm_LongRunHighGrayLevelEmphasis | 6.269058785 | 0.000000000727 | 0.6782424729 |
| glrlm_LongRunLowGrayLevelEmphasis | 0.2878321461 | 0.773551053 | 0.4404046059 |
| glrlm_LowGrayLevelRunEmphasis | -8.825833976 | 0 | 0.6743423001 |
| glrlm_RunEntropy | 20.17341766 | 0 | 0.8567675669 |
| glrlm_RunLengthNonUniformity | 22.98393503 | 0 | 0.8721690775 |
| glrlm_RunLengthNonUniformityNormalized | -6.595263622 | 0 | 0.648144659 |
| glrlm_RunPercentage | -4.151630658 | 0.000036442511 | 0.6161034878 |
| glrlm_RunVariance | 3.218658345 | 0.00136186275 | 0.6135356722 |
| glrlm_ShortRunEmphasis | -1.835454435 | 0.06686253127 | 0.5387675562 |
| glrlm_ShortRunHighGrayLevelEmphasis | 2.954210956 | 0.003220517536 | 0.6219766259 |
| glrlm_ShortRunLowGrayLevelEmphasis | -8.794975398 | 0 | 0.6597079042 |
| glszm_GrayLevelNonUniformity | 21.28690903 | 0 | 0.8607646384 |
| glszm_GrayLevelNonUniformityNormalized | -6.153259723 | 0.000000001191 | 0.5979295977 |
| glszm_GrayLevelVariance | 2.751503029 | 0.006057818783 | 0.6220143087 |
| glszm_HighGrayLevelZoneEmphasis | 3.925892482 | 0.000093393917 | 0.6464839229 |
| glszm_LargeAreaEmphasis | 2.766674185 | 0.005906076466 | 0.7464806929 |
| glszm_LargeAreaHighGrayLevelEmphasis | 8.263480508 | 0 | 0.8120380489 |
| glszm_LargeAreaLowGrayLevelEmphasis | 1.816506728 | 0.06998615 | 0.5696943923 |
| glszm_LowGrayLevelZoneEmphasis | -8.088577981 | 0 | 0.6630643677 |
| glszm_SizeZoneNonUniformity | 22.29104956 | 0 | 0.8996290933 |
| glszm_SizeZoneNonUniformityNormalized | -5.087895169 | 0.000000447685 | 0.6680869504 |
| glszm_SmallAreaEmphasis | 3.097514832 | 0.002023700497 | 0.582711118 |
| glszm_SmallAreaHighGrayLevelEmphasis | 5.391624627 | 0.000000093102 | 0.6780217591 |
| glszm_SmallAreaLowGrayLevelEmphasis | -4.177199914 | 0.000032918428 | 0.5445168792 |
| glszm_ZoneEntropy | 19.24128411 | 0 | 0.859251942 |
| glszm_ZonePercentage | -4.451895248 | 0.000009823169 | 0.5896555251 |
| glszm_ZoneVariance | 5.820169983 | 0.000000011269 | 0.7683178923 |
| ngtdm_Busyness | -0.8321557318 | 0.4057785819 | 0.4441917302 |
| ngtdm_Coarseness | -0.000002668360953 | 0.9999978716 | 0.5415076362 |
| ngtdm_Complexity | 6.107732195 | 0.000000001697 | 0.6463977907 |
| ngtdm_Contrast | -8.671702127 | 0 | 0.6646201302 |
| ngtdm_Strength | -5.988905092 | 0.000000003345 | 0.6882095811 |
